# Identification of transcriptional regulatory networks specific to pilocytic astrocytoma

**DOI:** 10.1186/1755-8794-4-57

**Published:** 2011-07-11

**Authors:** Hrishikesh Deshmukh, Jinsheng Yu, Jahangheer Shaik, Tobey J MacDonald, Arie Perry, Jacqueline E Payton, David H Gutmann, Mark A Watson, Rakesh Nagarajan

**Affiliations:** 1 Department of Pathology and Immunology, Washington University School of Medicine, 660. S. Euclid Ave. St. Louis, MO, 63110, USA; 2 Department of Pediatrics, Emory University, Atlanta, GA, 30322, USA; 3 Department of Neurology, Washington University School of Medicine, 660. S. Euclid Ave. St. Louis, MO, 63110, USA

## Abstract

**Background:**

Pilocytic Astrocytomas (PAs) are common low-grade central nervous system malignancies for which few recurrent and specific genetic alterations have been identified. In an effort to better understand the molecular biology underlying the pathogenesis of these pediatric brain tumors, we performed higher-order transcriptional network analysis of a large gene expression dataset to identify gene regulatory pathways that are specific to this tumor type, relative to other, more aggressive glial or histologically distinct brain tumours.

**Methods:**

RNA derived from frozen human PA tumours was subjected to microarray-based gene expression profiling, using Affymetrix U133Plus2 GeneChip microarrays. This data set was compared to similar data sets previously generated from non-malignant human brain tissue and other brain tumour types, after appropriate normalization.

**Results:**

In this study, we examined gene expression in 66 PA tumors compared to 15 non-malignant cortical brain tissues, and identified 792 genes that demonstrated consistent differential expression between independent sets of PA and non-malignant specimens. From this entire 792 gene set, we used the previously described PAP tool to assemble a core transcriptional regulatory network composed of 6 transcription factor genes (TFs) and 24 target genes, for a total of 55 interactions. A similar analysis of oligodendroglioma and glioblastoma multiforme (GBM) gene expression data sets identified distinct, but overlapping, networks. Most importantly, comparison of each of the brain tumor type-specific networks revealed a network unique to PA that included repressed expression of ONECUT2, a gene frequently methylated in other tumor types, and 13 other uniquely predicted TF-gene interactions.

**Conclusions:**

These results suggest specific transcriptional pathways that may operate to create the unique molecular phenotype of PA and thus opportunities for corresponding targeted therapeutic intervention. Moreover, this study also demonstrates how integration of gene expression data with TF-gene and TF-TF interaction data is a powerful approach to generating testable hypotheses to better understand cell-type specific genetic programs relevant to cancer.

## Background

Novel, albeit limited, insights into the molecular basis of human disease have resulted from the use of high-resolution, microarray-based platforms to comprehensively study complex diseases at multiple levels of the genetic program. For example, in central nervous system (CNS) tumors, array-based comparative genomic hybridization (aCGH) and DNA sequencing have led to the identification of tumor-associated chromosomal gains and losses and the corresponding specific gene mutations within these altered loci [1-3]. Gene expression-based studies have also profiled thousands of individual transcriptional units to uncover novel genes whose expression is de-regulated, concordant with other clinical features of these tumors, such as histological grade and patient survival [4-6]. The combined use of both technologies has identified individual genetic biomarkers associated with CNS tumorigenesis and tumor behavior, although few have yet resulted in specific strategies for targeted drug therapy [7].

The accumulation of 'genome-wide' data sets from CNS tumors is demonstrating that the processes of tumorigenesis and tumor progression likely involve the coordinated de-regulation of entire molecular networks, rather than single genes. Therefore, mutation, altered copy number, and abnormal expression of single genes observed to vary across individual tumors may be better viewed collectively to identify underlying commonalities at the level of molecular programs, based on known protein-protein interactions, canonical cell signaling pathways, and in silico transcriptional regulatory control predictions. As opposed to identifying single gene/protein targets for anti-cancer drug design, these networks establish broader, multi-target pathways for therapeutic intervention. The combination of higher-order DNA- and RNA-based microarray meta-analyses offers the potential to discover these therapeutically-relevant networks [8].

Pilocytic Astrocytomas (PAs) are World Health Organization (WHO) grade I glial CNS tumors, accounting for one-fifth of all central nervous system tumors. However, in young children and adolescents, PAs are the most common brain tumor. Compared to other high-grade CNS gliomas, such as Glioblastoma Multiforme (GBM), PAs are characterized by low cell proliferative indices, a biphasic histologic appearances, and microglial infiltration. PAs also lack the genetic alterations observed in high-grade glioma, and in general exhibit few molecular alterations at the DNA level. Previous studies have demonstrated occasional copy number changes involving chromosomes 6, 7, 8, and 11 [9,10]. In a recent study [1], we identified genome copy number alterations occurring in cerebellar PA using aCGH on patient-matched tumor and normal samples. However, as in other reported studies, only a limited number of consistent genetic alterations were identified.

Current treatments for these pediatric brain tumors include surgical resection, radiation, and chemotherapy. However, in young children, these treatments are associated with secondary damage to the developing brain, and result in long term neuropsychological and neuroendocrine dysfunction. In addition, since children with PA have good overall survival, it is imperative to identify the key genetic and growth control pathways de-regulated in the tumor and not the normal brain for future therapeutic drug design. Given this clinical imperative and the limited success in identifying therapeutic targets by conventional genomic analyses, we employed a higher-order, network-based analytical approach to discover subtle alterations in genetic pathways operative specifically in PA.

In the present study, we first identified PA-specific gene expression signatures in a meta-analysis of 74 PA microarray datasets. We then utilized the identity of these genes to construct a PA-specific gene network based on transcription factor gene regulatory interactions. Moreover, we identified relationships that are both common and specific to PAs compared to other cell lineage-related glial tumor types by creating similar gene regulatory networks from GBM and oligodendroglioma microarray datasets. Our approach has identified a unique genetic network in PA which may account for the novel phenotype of this brain tumor, and also demonstrates the general utility of using network-based approaches to analyze complex molecular profiling data from other tumor types.

## Methods

### Sample and Affymetrix GeneChip processing

All studies of de-identified, previously collected (existing) frozen human tumor tissue specimens were performed under an institutional reviewed and approved human studies protocol (WU HRPO #99-0573 and #04-0980). Frozen tumor tissues were sectioned and histologically reviewed to confirm pathological diagnosis and neoplastic cellularity (AP). Serial tissue sections was used to isolated tumor RNA using Trizol reagent (Invitrogen, Carlsbad, CA) and following the manufacturer's standard protocol. Resulting RNA was quantified by A260 and A280 readings using a Nanodrop spectrophotometer (Nanodrop Technologies, Wilmington DE) and qualitatively assessed using a BioAnalyzer 2100 and RNANanoChip assay (Agilent Technologies, Palo Alto, CA). All histology and nucleic acid extraction procedures were performed by the Siteman Cancer Center Tissue Procurement Core. Microarray data for 74 PA samples was generated with Affymetrix U133 Plus 2.0 arrays containing 54,675 probesets in the Siteman Cancer Center Molecular and Genomic Analysis Core (Washington University School of Medicine) starting with 1 μg of quality controlled total cellular RNA and using standard laboratory and manufacturer protocols.

### Data normalization and quality review

To facilitate comparison between multiple different datasets, meta-analysis was limited to the use of data generated using the Affymetrix GeneChip™ expression array platform. Data from 15 non-malignant frontal and prefrontal cortex brain tissue samples, hereafter referred to as non-malignant brain tissue samples, were obtained from ArrayExpress (E-TABM-20, E-TABM-84) [11,12]. Tumor datasets for GBM and oligodendroglioma were downloaded from GEO (GSE4412, GSE9385 and GSE4271 (GBM and oligodendroglioma) [5,6,13]) and The Cancer Genome Atlas (TCGA) [3]. For the GSE9385 dataset, which used Affymetrix GeneChip Exon arrays, non-malignant brain tissue samples that were also included in the dataset were used as the control set.

To perform inter-array comparisons and quality control, the raw data (i.e. CEL files) from each microarray were scaled to a target intensity of 1500 using the Affymetrix Expression Console (EC) version 1.1 (MAS 5.0 algorithm). Quality control review for all microarray data included percent present calls, RawQ, background, scaling factor, and 3'-5' ratios for GAPDH and beta-actin. Affymetrix control probes and probesets that were called "Absent" across all samples were removed. The MAS 5.0 data were z-normalized using the mean and standard deviation for each probe. Hierarchical agglomerative clustering was performed using both Spotfire's DecisionSite and Bioconductor software Unweighted pair-group method with arithmetic mean (UPGMA) was used as the clustering method, and Euclidian distance was used to calculate similarity.

Unsupervised hierarchical clustering was first performed on the entire 74 PA sample set. Clustering outliers and data sets which failed array quality control parameters were removed. The 15 non-malignant brain sample data sets were next added to the entire dataset, and z-normalization and hierarchical clustering was repeated. Tumor samples which segregated with non-malignant samples were also removed from further analysis. The final dataset consisted of 66 PA tumors and 15 non-malignant brain tissue samples. On retrospective review, the 8 PA tumor data sets excluded from analysis were confirmed to be initially misclassified based upon histological re-review of WHO grade I criteria for PA.

### Identification of gene expression signatures

**Figure 1:**
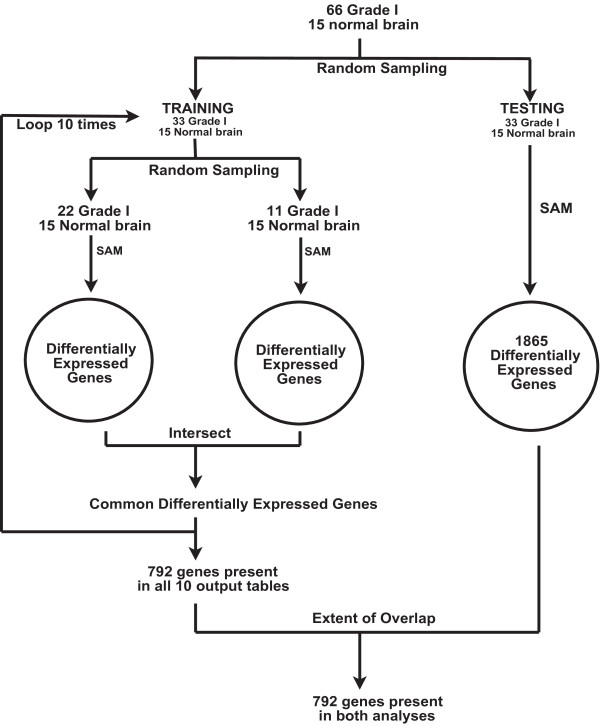
**SAM-based gene selection framework employed to identify differentially expressed genes in independent sets of pilocytic astrocytoma gene expression data**.

A gene expression signature that delineated PA from non-malignant brain tissue was identified using iterative and independent parallel data sets framework (Figure 1), based on the Significance Analysis of Microarrays (SAM) algorithm [14]. SAM was performed using Bioconductor with log2 transformed MAS 5.0 data filtered as described above. The 66 tumor samples were randomly divided into two groups of 33 samples. For the training arm of the protocol, the first 33 samples were randomly divided into two groups of 22 and 11. SAM analysis was conducted for each of these groups against the 15 non-malignant brain tissue samples. The minimum fold change threshold was defined as two-fold. Multiple testing correction was employed by setting the delta value to the highest value for which the 90th percentile of the FDR distribution remained equal to zero. Genes that were significant in both training samples groups were recorded and the samples were then randomized into two new training groups of 22 and 11 samples. This processed was repeated for 10 iterations. An output table was generated that consisted of genes that demonstrated consistent difference in expression in common between the two parallel training groups, in all 10 iterations of the training loop. The testing arm of the protocol then consisted of a single SAM analysis on the remaining 33 tumor samples of the entire 66 sample cohort. While it is possible that this approach may introduce bias and has the potential for false positives in the gene set because the test set was not resampled, the likelihood of this is low given that ALL genes identified in the training set were recapitulated in the test set, thus validating the robustness of the training loop. Furthermore, identified genes were further filtered through network construction as described in the next section.

To identify differentially expressed genes in other glial tumor histological types (GBM and oligodendroglioma), we employed the same 15 non-malignant brain tissue sample set (except for GSE9385 where the non-malignant controls provided in the data set were used), and a single SAM analysis on each of the GBM and oligodendroglioma datasets described above. Multiple datasets for GBM were available, and each dataset was analyzed separately using SAM to identify differentially-expressed genes.

### Network analysis to create a minimal gene regulatory network

**Figure 2:**
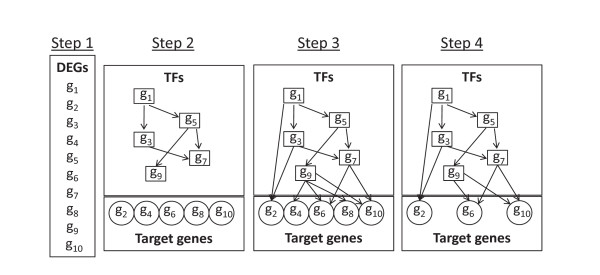
**Creation of a minimal transcriptional regulatory network**. Step 1: Differentially expressed genes (DEGs) are identified using SAM based selection. Step 2: Transcription Factors (TFs) and regulatory relationships (i.e. TF - TF) are identified among DEGs by using PAP. Step 3: A regulatory network is drawn by finding target genes regulated by TFs (i.e. TF - Target) from the DEG set using PAP. Step 4: A minimal network is generated by identifying targets regulated by more than one transcription factor.

The probe identifiers for the differentially expressed genes (DEGs) were mapped to Entrez Gene IDs using Affymetrix-provided Accession Numbers and annotations available from NCBI's Entrez Gene database, and Entrez Gene IDs were used to identify transcription factors (TFs) from TRANSFAC 11.4 and JASPAR databases. The Promoter Analysis Pipeline (PAP) [15] was next employed to construct the TF-TF network (Figure 2-step 2) and the TF-target network (Figure 2-step 3). A p-value cutoff of 0.005 was used to select TF-target interactions from PAP. A regulatory network was defined as a cumulative representation of all interactions between TFs and their known target genes, one or both of which was represented in the DEG list. In this arrangement, therefore, a regulatory network may encompass many branching sub-networks, even if only one major network is of interest based upon gene expression data. Multiple studies have found that TFs work in conjunction with each other to regulate genes; thus, this information was incorporated into the regulatory network by finding targets that are commonly regulated by more than one TF (Figure 2-step 4). This network is called a minimal network (i.e. the resulting network where each target gene is regulated by at least two TFs). The differential expression analysis provided quantitative data on over-expression or under-expression of a gene in PA samples when compared to non-malignant brain tissue samples. When overlaid on the regulatory network, this information provided the difference in expression of a gene in response to difference in expression of another gene. Finally, the hypergeometric distribution test was used to identify overrepresented transcriptional regulatory pathways predicted by the minimal network gene list [16].

The same approach was used to create a minimal network for the oligodendroglioma dataset. Multiple GBM datasets were used to identify differentially expressed genes, and minimal networks were created as described above. These individual GBM networks were pooled together by taking the union of all relationships for each GBM dataset and this consolidated set was used to create the composite GBM minimal network. Finally, while this approach is limited to constructing a network using only those TFs that have demonstrable differential expression and may thus not identify all TFs and TF-target interactions, we chose to employ the above algorithm given the experimental data sets that were employed for network construction and the desire to construct a more robust, though perhaps incomplete, network.

### Identification of relationships in common with tumor types and specific to PA

The pair-wise relationships generated as a result of network analysis of PA and other glial tumor types allowed the identification of relationships common across tumor types as well as those specific to PA. A relationship was defined as common when a relationship between two genes that was identified in PA (based upon the DEG list) was also present in one or more other tumor types (GBM or oligodendroglioma). A relationship was defined as specific to PA when the relationship was present between two genes in PA but was not present in any of the other glial tumor types.

## Results and Discussion

### Identification of PA-specific gene expression patterns

We used a relatively large sample set, divided into two independent sets for SAM analysis (Figure 1) to identify differentially-expressed gene transcripts which were recurrently represented in PA samples compared to non-malignant cortical brain tissue samples. Using this framework, we were able to identify genes scored as 'significant' (FDR = 0% by SAM) across multiple iterative analyses using random sampling of the first PA expression dataset, and then confirm that the signature identified was also represented in a second, independent set of tumor samples. This method was modeled on existing supervised methods, including Support Vector Machines and Artificial Neural Networks [17,18], and facilitated the reliable identification of differentially-expressed genes in the absence of large independent datasets not currently available for PA. One recognized limitation of this approach is that the same reference set of 15 non-malignant brain tissue samples was used in all of the SAM analyses. Ideally, if such material or data were available, it would have been desirable to perform comparative analysis using an independent set of non-malignant tissue samples, as was done for the PA samples themselves. Second, although PAs, GBMs, and oligodendrogliomas could have been directly compared against each other, differentially expressed genes may represent differences in cells of origin of each tumor type rather than be important for initiation, maintenance, and/or growth/metastasis of that tumor type. Because cell of origin is not known for these tumor types, we could not use in vivo or in vitro derived normal cells as a comparison. Therefore, non-malignant brain tissue, which is a mix of cells of origin, was utilized to compare against each tumor type. This approach identified 792 genes that showed consistent differential expression between the reference tissue data and multiple sets of PA tumors in the training data set, and these 792 genes were also differentially expressed in the test data set.

### Use of network analyses to create a minimal gene regulatory network

Multiple studies have identified individual gene transcripts that are differentially expressed in PA relative to other malignant and non-malignant cell populations [4]. In the current study, we identified 792 genes using this larger sample set. However, to impart higher-order biological significance to the list of genes we identified and to select groups of genes from this list that may, as a network, provide additional insights into the unique clinical or molecular features of PA, we sought to identify gene regulatory relationships that comprise networks unique to this common pediatric astrocytoma histologic subtype. This approach is distinct from other approaches that define molecular networks based on direct or indirect gene-gene interactions [19,20]. Many previous approaches use existing literature annotation at the gene and cell biology level, including canonical pathways, and heavily depend on human curation of the biological knowledge in the published literature [21]. A recent study used the Algorithm for the Reconstruction of Accurate Cellular Networks (ARACNe), an information-theoretic approach, to infer transcriptional networks underlying mesenchymal transformation of high grade gliomas [22-24]. While ARACNe calculates mutual information between each pair of genes using expression information and identifies TF-target interactions using annotations from TRANSFAC and Gene Ontology, it does not employ statistical approaches to predict TF-target interactions using TF position weight matrices and phylogenetic footprinting. Therefore, in the current analysis, we have implemented a novel analytical method previously developed and described, Promoter Analysis Pipeline (PAP) [25,26]. PAP exhaustively searches for TF binding sites in the promoter regions of genes, identifies over-represented binding sites, and predicts genes in the genome that are most likely regulated by a set of TFs. PAP is available to the community through our website (http://bioinformatics.wustl.edu/webTools/PromoterAnalysis.do).

We melded *in silico *genome-wide PAP-derived predictions together with an empirically-determined list of differentially-expressed gene sets identified by microarray expression analysis to identify transcription factors (TFs) that regulate other transcription factors (Figure 2, step 2). Subsequently, the network was expanded to include TF-target gene relationships (Figure 2, step 3), and was then pruned to include only those genes regulated by more than one TF (Figure 2, step 4). This output, termed the minimal network, represents both the TF cascade and core TF-target gene relationships that are predicted to operate in the disease under study.

**Figure 3:**
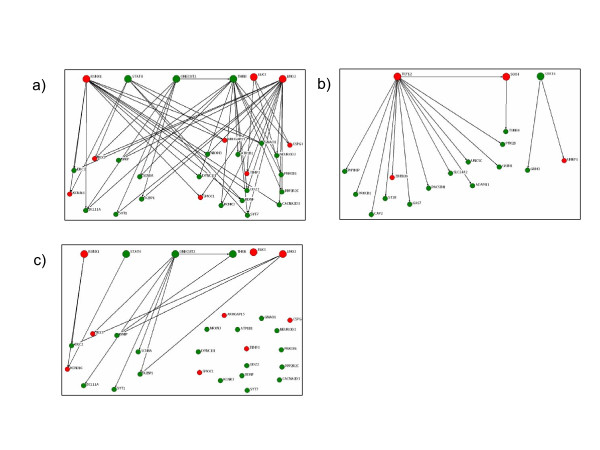
**Minimal transcriptional regulatory networks in gliomas**. (A) Minimal network for pilocytic astrocytoma. Transcription factors (TF) are shown by large solid circles, target genes are shown by small solid circles. Interactions between TF-TF and TF-target are shown by directed edges. Genes overexpressed in PA relative to non-malignant brain tissue are shown in red, while genes whose expression is lower in PA are shown in green. (B) Similar relationships depicted in the minimal network assembled from the oligodendroglioma gene expression data set. (C) Relationships that are unique to PA as compared to the minimal network assembled for GBM.

From the 792 'significant genes' first identified by SAM analysis of which 197 genes were up-regulated and 595 genes down-regulated (Additional file 1 STable 2), the minimal PA network is composed of a set of six core TFs (STAT4, RUNX1, ELK3, LMO2, THRB, and ONECUT2), twenty-four corresponding target genes, and a total of fifty-five relationships between them (Figure 3A; the complete PA network predicted is available as Additional file 2, SFig 1). Although the way in which these predicted gene interactions influence PA pathogenesis is not immediately obvious, it is worth noting that the functions of proteins encoded by several of these individual transcripts have been implicated in a variety of other tumor types, while RUNX1 and SMOC1 may be more specifically related to astrocytoma biology [27,28].

### Identification of PA-specific and common glial transcriptional networks

To better understand whether the PA transcriptional network was specific to this glial tumor type, we generated analogous networks for oligodendroglioma and glioblastoma multiforme tumors, and these were compared to the PA minimal network. The comprehensive oligodendroglioma network that was created through a similar analysis of oligodendroglioma gene expression data sets and PAP-generated interactions (step 3 in Figure 2) is shown in Figure 3B. There are sixteen gene regulatory interactions among the three TFs (STAT4, TCF12, and SOX4) and fifteen targets. Compared to the assembled PA minimal network, the oligodendroglioma network is less elaborate, probably due to the more limited gene expression data set available for its generation. Importantly, there were no genes or relationships shared between the PA and oligodendroglioma networks. The lack of overlap between PA and oligodendroglioma networks contrasts to other data that suggests the presence of oligodendroglial-like features in PA tumors [29] and the expression of oligodendroglial-specific transcription factors, like SOX10, in astrocytic tumors [30]. However, common pathological features between PA and oligodendroglioma do not necessarily imply a common histogenesis, nor do they preclude the notion that these two glial tumor types are distinct pathological and genetic entities.

By comparison, the minimal network assembled for glioblastoma multiforme included 2,933 total genes, 194 TFs, and 25,038 gene regulatory interactions. This result no doubt reflects the complexity and heterogeneity of the GBM genome [3] and is consistent with the network generated by ARACNe using high grade glioma expression data sets [24]. All relationships identified in the oligodendroglioma network (Figure 3B) were also represented in the GBM network, suggesting greater similarity between these two tumor types. Of note, master regulators (C/EBP beta and delta, STAT3, FOSL2, RUNX1, and bHLH-B2) with the exception or bHLH-B2 operating in high grade gliomas that were identified by Carro, et al [24], were all found in our GBM network as well (Additional file 3, STable 1). In contrast, among the fifty-five relationships in the PA minimal network, forty-one were shared between PA and glioblastoma multiforme, but 13 were specific to PA (Figure 3C). There were five individual transcription factor interactions (RUNX1, STAT4, THRB, LMO2, and ELK3) shared between PA and GBM, but only one TF interaction, ONECUT2, was specific to PA. ONECUT2 is a bipartite DNA-binding domain-containing homeobox gene highly expressed in the developing mouse nervous system during embryogenesis, and has been shown to be important for regulating neural differentiation in Drosophila [25]. Furthermore, ONECUT2 is often methylated or silenced in human cancer, and may be amenable to methylation-directed therapies [26].

## Conclusions

In the current study, we employed only TF-gene interactions to assemble predicted regulatory networks. The use of this methodological approach illustrates how traditional, microarray-based gene expression data can be integrated with a novel TF-gene interaction database generated from primary genome sequence analysis to reveal higher-order, cell-specific transcriptional regulatory networks. The approach detailed in this report has significant advantages over conventional microarray methods aimed at identifying 'significant' differential gene expression. First, many low-grade tumors, like PAs, harbor few obvious genetic alterations (e.g., oncogenic mutations, translocations), and the critical changes that facilitate tumorigenesis and continued growth may reflect de-regulated gene expression patterns. These latter alterations may result from "genetic re-programming" due to changes in TF expression, and would only be uncovered by examining the impact of these changes in the context of coordinated gene expression patterns. Second, the ability to extract gene expression patterns is often limited by the depth of the current scientific literature knowledgebase, and can be arduous and unenlightening, particularly when candidate gene lists are large (e.g., 792 genes in this study). The use of the PAP method represents a more robust approach for identifying coordinated patterns of gene transcription by coupling downstream TF target expression levels with a corresponding TF-gene interaction network based on sequence-predicted interactions. Lastly, direct measurements of differential TF gene expression are difficult, particularly by relatively insensitive microarray-based methods, owning to the inherent low levels of TF gene expression and the effect that even small changes in TF levels may have on downstream targets and cell phenotype. Further application of the approach presented here could involve the use of additional genome annotation derived from TF protein interaction databases, literature-based gene-gene interaction data, and miRNA-gene target data. For example, patterns of coordinated gene expression derived from experimental microarray-based data could be superimposed upon gene expression regulation modeled as a linear combination of probabilistic contributions from individual TFs and miRNAs using multiple regression methods.

## Abbreviations

aCGH: array-based Comparative Genomic Hybridization; PA: Pilocytic Astrocytoma; WHO: World Health Organization; GBM: Glioblastoma Multiforme; TCGA: The Cancer Genome Atlas; UPGMA: Unweighted pair-group method with arithmetic mean; SAM: Significance Analysis of Microarrays; DEGs: Differentially Expressed Genes; TFs: Transcription Factors; PAP: Promoter Analysis Pipeline.

## Competing interests

The authors declare that they have no competing interests.

## Authors' contributions

HD conducted the network analyses described in this manuscript and assisted with manuscript preparation. JY, JS, and JP provided additional input on analyses and assisted with manuscript preparation. TM, AP, and DG provided tissue samples, pathological review, and input into manuscript preparation. MW supervised conduct of microarray studies and data generation, and assisted with manuscript authoring. RN provided study design, project oversight, and final manuscript review. All authors read and approved the final manuscript.

## Pre-publication history

The pre-publication history for this paper can be accessed here:

http://www.biomedcentral.com/1755-8794/4/57/prepub

## Supplementary Material

Additional file 1 **The 792 gene set that is differentially expressed in PAs along with each gene's expression differences when compared to the control are shown**. Click here for file

Additional file 2 **PA network obtained as a result of step 3 in Figure 2**. Transcription factors (TF) are shown by large solid circles, target genes are shown by small solid circles. Interactions between TF-TF and TF-target are shown by directed edges. Genes overexpressed in PA relative to non-malignant brain tissue are shown in red while genes whose expression is lower in PA are shown in green. Click here for file

Additional file 3 **Gene regulatory relationships in glioblastoma multiforme minimal network**. The Transcription Factors (TFs) and respective relationships between their targets are shown. Click here for file
