# Supplementary figures and images for: Identification of transcriptional regulatory networks specific to pilocytic astrocytoma
Source: BMC Med Genomics. 2011 Jul 11;4:57. doi: 10.1186/1755-8794-4-57 (PMC3200988; doi:10.1186/1755-8794-4-57)

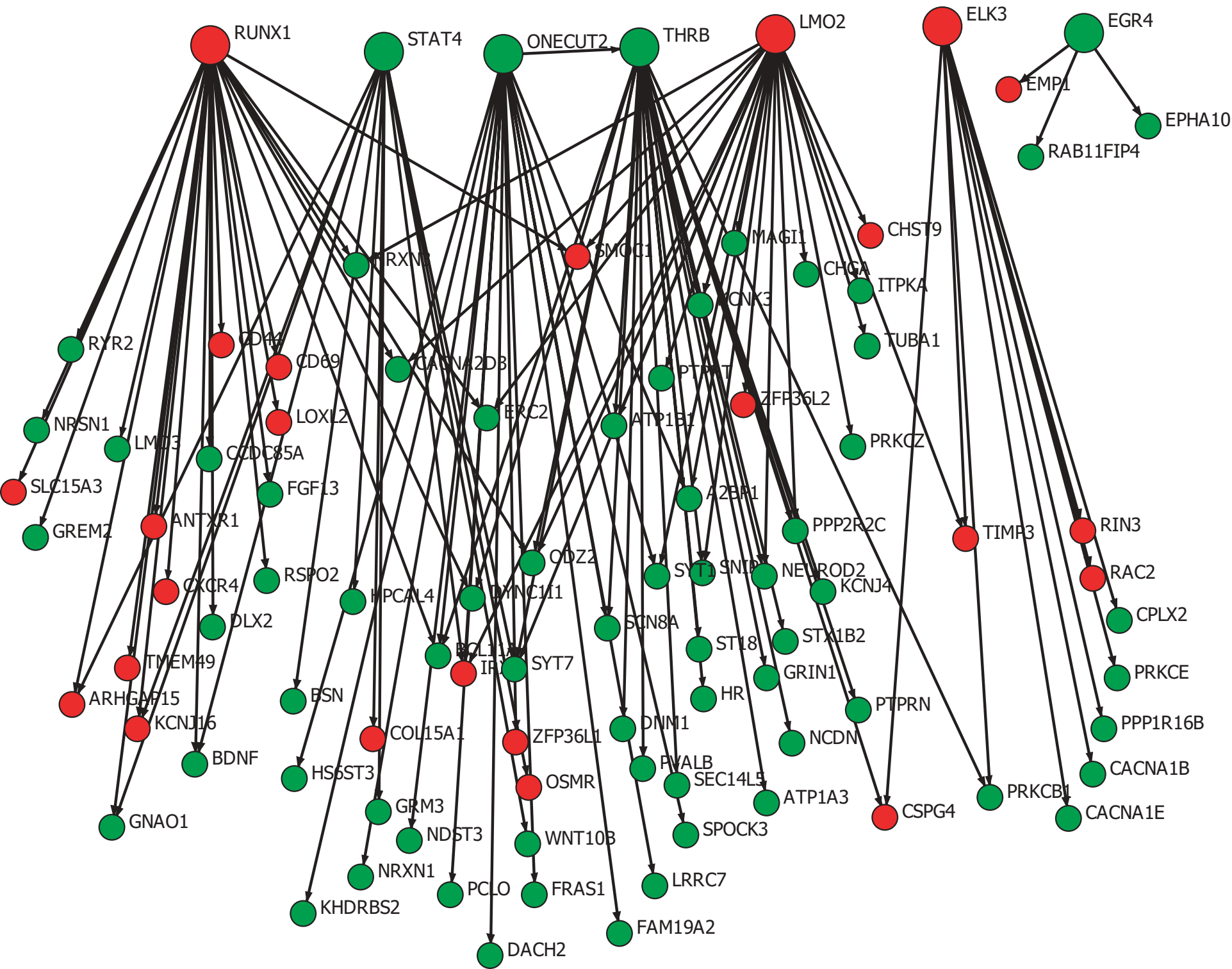

Supplement: Additional file 2 — PA network obtained as a result of step 3 in Figure 2. Transcription factors (TF) are shown by large solid circles, target genes are shown by small solid circles. Interactions between TF-TF and TF-target are shown by directed edges. Genes overexpressed in PA relative to non-malignant brain tissue are shown in red while genes whose expression is lower in PA are shown in green. [file 1755-8794-4-57-S2.pdf]
